# Vismodegib: First-in-Class Hedgehog Pathway Inhibitor for Metastatic or Locally Advanced Basal Cell Carcinoma

**DOI:** 10.6004/jadpro.2014.5.4.7

**Published:** 2014-07-01

**Authors:** Nancy M. Nix, Olivia Burdine, Makeda Walker

**Affiliations:** From St. Joseph’s/Candler Health System–South Carolina Cancer Specialists, Hilton Head, South Carolina; University of Georgia, Athens, Georgia; Mercer University, Atlanta, Georgia

Skin cancer is the most common cancer type in the United States, affecting more than 3 million people nationally (Cirrone & Harris, 2012). Basal cell carcinoma (BCC) and squamous cell carcinoma are the two main types of nonmelanoma skin cancer, while the more serious form, melanoma, is in its own category. An estimated 80% of nonmelanoma skin cancers are BCCs (Sekulic et al., 2012). Features such as stage, cancer history, and extent of disease determine BCC prognosis (Crowson, 2006). Basal cell carcinoma rarely metastasizes but is capable of local tissue destruction and commonly occurs in the geriatric patient population.

The initial presentation of BCC is generally a pigmented nodule on the head or neck. Risk factors for BCC include chronic sun exposure; fair complexion; male gender; history of nonmelanoma skin cancer; family history of skin cancer; history of chronic arsenic exposure; or chronic immunosuppression, such as in lymphoma, transplant, or HIV-positive patients (Crowson, 2006).

Topical agents such as imiquimod or fluorouracil (5-FU) can be used in low-risk superficial BCC (Fellner, 2012). However, surgery is the initial treatment for BCC. Complete excision of the tumor is preferred. Adjunctive radiation therapy is needed if the tumor margins were not adequately cleared during surgery. If surgery is impossible due to the location of the cancer or other complications, systemic chemotherapy agents such as 5-FU and cisplatin can be considered alternative options.

With the approval of vismodegib (Erivedge), patients with metastatic or locally advanced BCC who are unable to undergo surgery now have a treatment alternative that may provide a beneficial outcome. Vismodegib is approved for metastatic BCC or locally advanced BCC that has recurred following surgery, or for those who are not candidates for surgery and who are not candidates for radiation therapy (Fellner, 2012).

## Pharmacology

Vismodegib, which exerts its effects on the hedgehog (Hh) pathway, was approved in January 2012 by the US Food and Drug Administration (FDA) for monotherapy in the treatment of unresectable/malignant and advanced BCC (Fellner, 2012). This drug was discovered via experimentation on fruit flies, which showed that the hedgehog pathway is important in regulating growth, embryogenesis, tissue homeostasis, and many other functions important in development (Cirrone & Harris, 2012). Three main hedgehog pathway ligands have been identified as key factors in BCC: sonic hedgehog ligand (SHh), Indian hedgehog ligand (IHh), and desert hedgehog ligand (DHh). The inappropriate activation of the hedgehog pathway via these ligands is believed to be the etiology of BCC in many individuals (Sandhiya, Melvin, Kumar, & Dkhar, 2013).

The role of hedgehog ligands and pathway stimulation in BCC is theorized to lie in the activation of a 12-transmembrane domain protein (Patch1, abbreviated PCTH1) that is usually present during the telophase of the cell cycle (Ruch & Kim, 2013). This protein normally regulates cell entry activity of smoothen (SMO), a 7-transmembrane protein receptor, by inhibiting its entry. When hedgehog ligands bind to the 12-transmembrane domain protein (PCTH1), it causes the loss of inhibition of smoothen which allows it to enter the cell (Cirrone & Harris, 2012). This leads to smoothen activation, which further stimulates downstream pathways, resulting in increased cell proliferation. Smoothen activation and subsequent signaling is believed to be the key problem that causes BCC (Ruch & Kim, 2013).

Vismodegib antagonizes smoothen by binding to and inhibiting its movement inside the cell while also preventing transmission of signals. By antagonizing smoothen, vismodegib is capable of preventing cell proliferation caused by either a genetic mutation or an environmentally induced factor. A potential downside to the mechanism of vismodegib is the possibility for resistance to occur further downstream from the SMO receptor (Cirrone & Harris, 2012).

## Clinical Trials

A total of 68 participants with refractory, locally advanced, or metastatic solid tumors, 33 of whom had advanced BCC, were enrolled in a phase I trial investigating the hedgehog pathway inhibitor vismodegib. The objectives consisted of determining tolerability and safety, identifying maximum dose, discovering dose-limiting toxicities, assessing pharmacokinetics, and determining a phase II dose and schedule regimen. All participants in the study were at least 18 years of age, with the median age being 54. Exclusions from the study included patients with conditions such as major organ dysfunction, prolonged QT interval, and active infection requiring IV antibiotics (LoRusso et al., 2011). Vismodegib was well tolerated, and no maximum tolerated dose or dose-limiting toxicity was established. When the dosing regimen was assessed, multiple daily dosing of 150, 270, or 540 mg failed to show a difference in steady-state concentration. Due to this fact, the recommended dose of vismodegib was established at 150 mg daily (LoRusso et al., 2011).

Tumor assessment was performed via radiologic testing or physical examination to evaluate response rate. Disappearance of the tumor observed by physical exam was labeled as a complete response, and decrease in 50% or more of the tumor was considered a partial response. Of the 33 patients with BCC, 17 experienced a partial response, 2 experienced a complete response, 10 exhibited stable disease, and 4 showed signs of disease progression (LoRusso et al., 2011). Due to the positive response rates of vismodegib a phase II trial was initiated.

The phase II trial entitled ERIVANCE BCC enrolled 104 patients with either locally advanced or metastatic BCC. The primary efficacy endpoint of objective response rate was measured using Response Evaluation Criteria in Solid Tumors (RECIST) guidelines, version 1.0. Vismodegib proved to decrease cancerous lesions by 30% in the participants suffering from metastatic BCC (95% confidence interval [CI] = 16%–48%; *p* = .001). An even greater response was seen in patients suffering from advanced BCC: 43% (95% CI = 31%–56%; *p* < .001) with 13 complete responses (Sekulic et al., 2012). In both cohorts, the median duration of response was 7.6 months.

## Adverse Effects

According to the phase I trial, the most common adverse effects experienced by patients taking vismodegib were muscle spasms, fatigue, alopecia, dysgeusia, and nausea. Although minimal, other side effects noted were hyponatremia, pyelonephritis, anxiety, and hyperglycemia. In addition, vismodegib also caused cough, back pain, and vomiting in more than 10% of participants (Fellner, 2012).

The phase II trial of vismodegib reported that the most common reason for discontinuation was progression of disease. Of 104 patients, 26 experienced serious adverse events that included unknown cause of death, hypovolemic shock, myocardial infarction, meningeal disease, and ischemic stroke. During the study, it was reported that all patients experienced at least one adverse effect, including but not limited to muscle spasm, alopecia, decrease in weight, nausea, diarrhea, dysgeusia, and decrease in appetite. Showing a similar side-effect profile to that seen in the phase I study, the reason for patient deaths observed in the phase II study `remains unknown and was considered by trial investigators to be unrelated to vismodegib (Sekulic et al., 2012).

## Implications

The FDA approved vismodegib as the first hedgehog pathway, SMO inhibitor, which has proved to be a well-tolerated alternative for patients with metastatic or advanced BCC. The National Comprehensive Cancer Network (NCCN) guidelines addressing BCC (version 2.2013) recommend vismodegib as an alternate therapy for those who have failed or have been unable to try other options (NCCN, 2013). The approval of this novel antitumor agent makes it essential that those prescribing and dispensing vismodegib are aware of pertinent information vital to response rate and efficacy (Sekulic et al., 2012).

Vismodegib is provided as a 150-mg capsule intended to be taken by mouth once daily. It may be taken without regard to meals but should not be split, opened, or altered in any way prior to administration. Vismodegib has a pregnancy category D rating and is not indicated for use during pregnancy because its mechanism of action may result in serious fetal harm. Pediatric indications for vismodegib do not exist, and due to adverse effects on developing bone growth, use in the pediatric population is not recommended. Vismodegib has not been tested in patients with renal and/or hepatic deficiencies, rendering dosage information unavailable for these populations. Since vismodegib is a minor substrate of p-glycoprotein as well as cytochrome P450 3A4 and 2C9 isoenzymes, careful monitoring of therapeutic effect should be considered if the concurrent use of inducers or inhibitors of these enzymes is required (Fellner, 2012).

## Summary

Treatment options for those with metastatic and advanced BCC unable to undergo surgery are limited. The NCCN has updated the BCC guidelines to include vismodegib as an option for high-risk patients who have exhausted other options. Although vismodegib produced many side effects in its clinical trials, reduction in tumor size and potential for complete response make it an attractive therapy option for those ineligible for surgery. Vismodegib is breaking down barriers in BCC and has the potential to leave its imprint on the world of oncology while paving the way for drugs with a similar mechanism of action. 
